# DRG payment for major pancreatic surgery: analysis of resource consumption and suggestions from a tertiary hospital in China

**DOI:** 10.3389/fpubh.2024.1437272

**Published:** 2024-09-18

**Authors:** Rui Hou, Xiaokun Liu, Jingya Zhou, Taiping Zhang, Weibin Wang, Weiguo Zhu

**Affiliations:** ^1^Department of General Surgery, State Key Laboratory of Complex Severe and Rare Diseases, Peking Union Medical College Hospital, Chinese Academy of Medical Sciences and Peking Union Medical College, Beijing, China; ^2^Department of Medical Insurance Management, State Key Laboratory of Complex Severe and Rare Diseases, Peking Union Medical College Hospital, Chinese Academy of Medical Sciences and Peking Union Medical College, Beijing, China; ^3^Department of Medical Records, State Key Laboratory of Complex Severe and Rare Diseases, Peking Union Medical College Hospital, Chinese Academy of Medical Sciences and Peking Union Medical College, Beijing, China; ^4^Department of Primary Care and Family Medicine, State Key Laboratory of Complex Severe and Rare Diseases, Peking Union Medical College Hospital, Chinese Academy of Medical Sciences and Peking Union Medical College, Beijing, China

**Keywords:** DRG, pancreatic surgery, pancreaticoduodenectomy, robotic-assisted surgery, coefficient of variation

## Abstract

**Aim:**

To investigate the cost homogeneity within the Diagnosis-Related Group (DRG) “major operation of pancreas and liver, with general complications or comorbidities” (HB13), its cost-influencing factors, and to propose suggestions for better grouping efficacy.

**Methods:**

Medical and insurance settlement data of inpatients covered by the DRG payment system at the author’s institution were collected from March 15, 2022 to December 31, 2023. The cost homogeneity of group HB13 was assessed using the coefficient of variation (CV). Clinical factors that may have an impact on hospitalization cost for patients undergoing pancreatic surgery were identified through a semi-structured interview administered to the pancreatic surgeons in author’s department, their significance was analyzed using multiple linear regression, along with their impact on the cost of different service categories. A proposal to subdivide HB13 was made and evaluated by CV and t-test.

**Results:**

The CV of the HB13 group was 0.4. Robotic-assisted surgery and pancreaticoduodenectomy were two independent factors that significantly affected the total cost. Patients undergoing robotic-assisted surgery have an average increase of 41,873 CNY in total cost, primarily derived from operation fee. Patients undergoing pancreaticoduodenectomy have an average increase of 37,487 CNY in total cost, with significant increases across all service categories. HB13 was subdivided based on whether pancreaticoduodenectomy was performed. The newly formed groups exhibited lower CVs than the original HB13.

**Conclusion:**

The cost homogeneity of HB13 was lower than that of other DRG groups in author’s department. It is recommended to introduce a supplementary payment for patients requiring robotic-assisted surgery, to guarantee their access to this advanced technology. It is recommended to establish a new group with higher payment standard for patients undergoing pancreaticoduodenectomy. A tiered CV criterion for the evaluation of grouping efficacy is recommended to increase intra-group homogeneity, facilitating a better allocation of health insurance funds, and the prevention of unintended negative outcomes such as service cuts and cherry-picking.

## 1 Introduction

Since the initiation of reform and opening-up policies, China’s healthcare sector has made remarkable achievements, evidenced by a near-79-year life expectancy and the establishment of a universal health insurance system covering the entire population (1, 2). However, with China’s aging population and its increasing demand for medical services, the country’s health insurance system faces ever more serious challenges. Over past years, China’s health insurance payment employed the traditional form of “fee-for-service” (FFS), which, while simple and easily manageable, inevitably leads to several negative effects such as over-treatment, imbalanced allocation or wastage of medical resources, and ultimately results in unreasonable increases in medical expenses (3).

Facing these problems, China introduced and began to adopt Diagnosis-Related Groups (DRG) payments for inpatient services (3, 4). DRG system, originating in the U.S in the 1970s and now widely utilized in western countries, classifies hospital cases into diagnosis-related groups of patients with similar clinical characteristics and comparable costs, and pays hospitals a flat fee for each group that reflects national average costs for patients in that grouping (5). It has been proven to neutralize the negative effects of FFS, facilitate the standardization of clinical pathways, increase transparency and productivity of hospital activities, and prompt medical institutions to provide homogeneous and effective medical services at lower costs (5–7). To be a well-functioning and sustainable payment system, DRG necessitates a scientific grouping scheme and accurate payment; otherwise, it may produce adverse effects such as upcoding, cherry-picking, cutting services and reducing quality (5, 8, 9).

**Figure 1:**
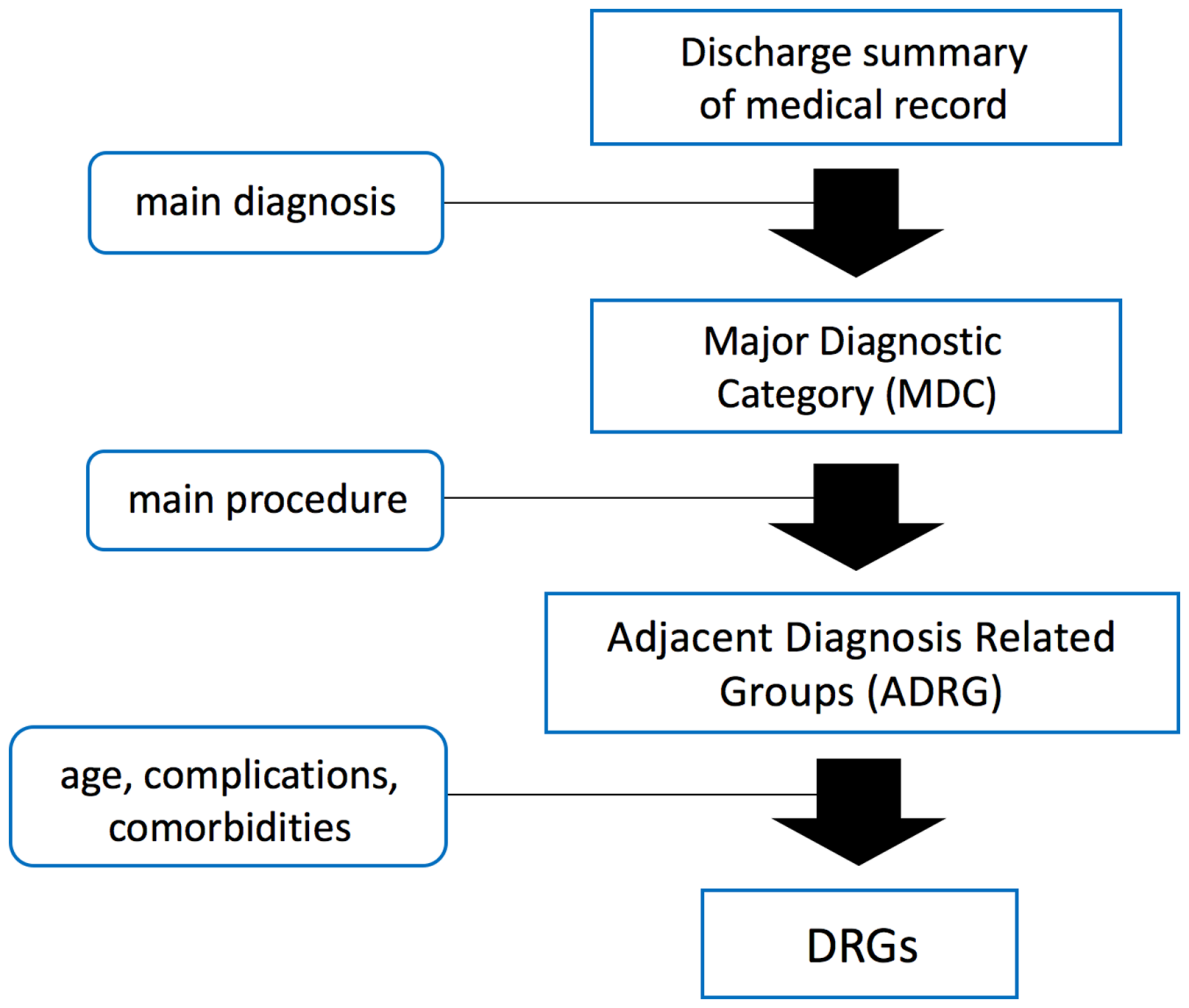
Basic grouping principle of CHS-DRG.

Combining western models with domestic context and data, China released its own DRG grouping scheme and payment standard (CHS-DRG) in 2019 (4, 8). Its basic principle of grouping is shown in Figure 1 (7). Each DRG group encompasses cases that share similar clinical characteristics, treatment trajectories, and comparable resource consumption. The grouping efficay is evaluated by the coefficient of variation (CV, SD divided by mean) of hospitalization cost, where a higher CV indicates lower intra-group homogeneity and less similarity among the cases within the group. The current grouping criterion for CHG-DRG is CV less than 1 for each group. The payment standard of each group is determined by multiplying the unit cost by the group’s relative weight (RW), which reflects the relative resource consumption among different DRG groups. Higher RW signifies a higher average hospitalization cost for the corresponding group (3, 8). Since the implementation of CHS-DRG, concerns, even criticisms have been heard regarding some inappropriate grouping schemes and inaccurate payment which exert negative effects on clinical activities. But most of them stem from experiences and individual cases, without verification by statistical analysis of the cost and influencing factors. Based on the data from the author’s institution, we conducted a statistical investigation into the hospitalization cost and its influencing factors of the cases within the HB13 group (major operation of pancreas and liver, with general complications or comorbidities). The aim of the present study is to evaluate the efficacy of CHS-DRG grouping for pancreatic surgery cases, identify clinical factors that impact hospitalization costs, and propose recommendations for grouping schemes and payment.

## 2 Data source and method

### 2.1 Data source

The data consisted of medical records and insurance settlement data of all inpatients covered by national basic health insurance from the Department of General Surgery of author’s institution. The time span of the included cases is from Mar. 2022 (when Beijing officially began DRG payment) to Mar. 2024. The specific data types included were: admission date, length of hospital stay, age, gender, primary diagnosis, secondary diagnosis, primary procedure, secondary procedure, DRG grouping, RW, total hospitalization cost, cost for each service category (medical consumables, operation, drugs, laboratory tests, radiology, therapy, others), and other key clinical characteristics. The data were jointly provided by the Department of Medical Records and the Department of Health Insurance of author’s institution. Preprocessing was conducted to address missing or inaccurate data fields and to ensure the linkage between medical record data and insurance settlement data.

### 2.2 Study method

The hospitalization cost of each DRG was described using mean, extremes and standard deviation (SD). CV of cost in each DRG was calculated to describe the intra-group homogeneity.

A semi-structured interview was conducted with pancreatic surgeons at the author’s department who have at least 3 years of working experience to identify clinical factors that might contribute to increased hospitalization costs for patients undergoing pancreatic surgery. The interview consisted of 3 questions: 1. Do you think the current grouping scheme of pancreatic surgery patients meets the needs of clinical practice? 2. Do you think the current payment standard for pancreatic surgery patients is appropriate? 3. Which clinical factors may significantly increase the hospitalization cost of pancreatic surgery patients? During the interviews, a neutral stance was maintained, and the answers of the interviewees were noted, from which the key information and words were extracted. Results were presented as frequencies.

Based on the results of the interviews, multiple linear regression analysis was conducted to identify the clinical factors that significantly influence the total hospitalization cost and their impact across different service categories. Suggestions for better grouping efficacy were then proposed and validated through t-tests. All statistical analyses and graphical presentations were performed using SPSS and GraphPad software. Differences were considered statistically significant when *p* < 0.05 (*p* < 0.05: *; *p* < 0.005: **; *p* < 0.001: ***).

## 3 Result

### 3.1 Descriptive analysis of medical and insurance settlement data

**Figure 2:**
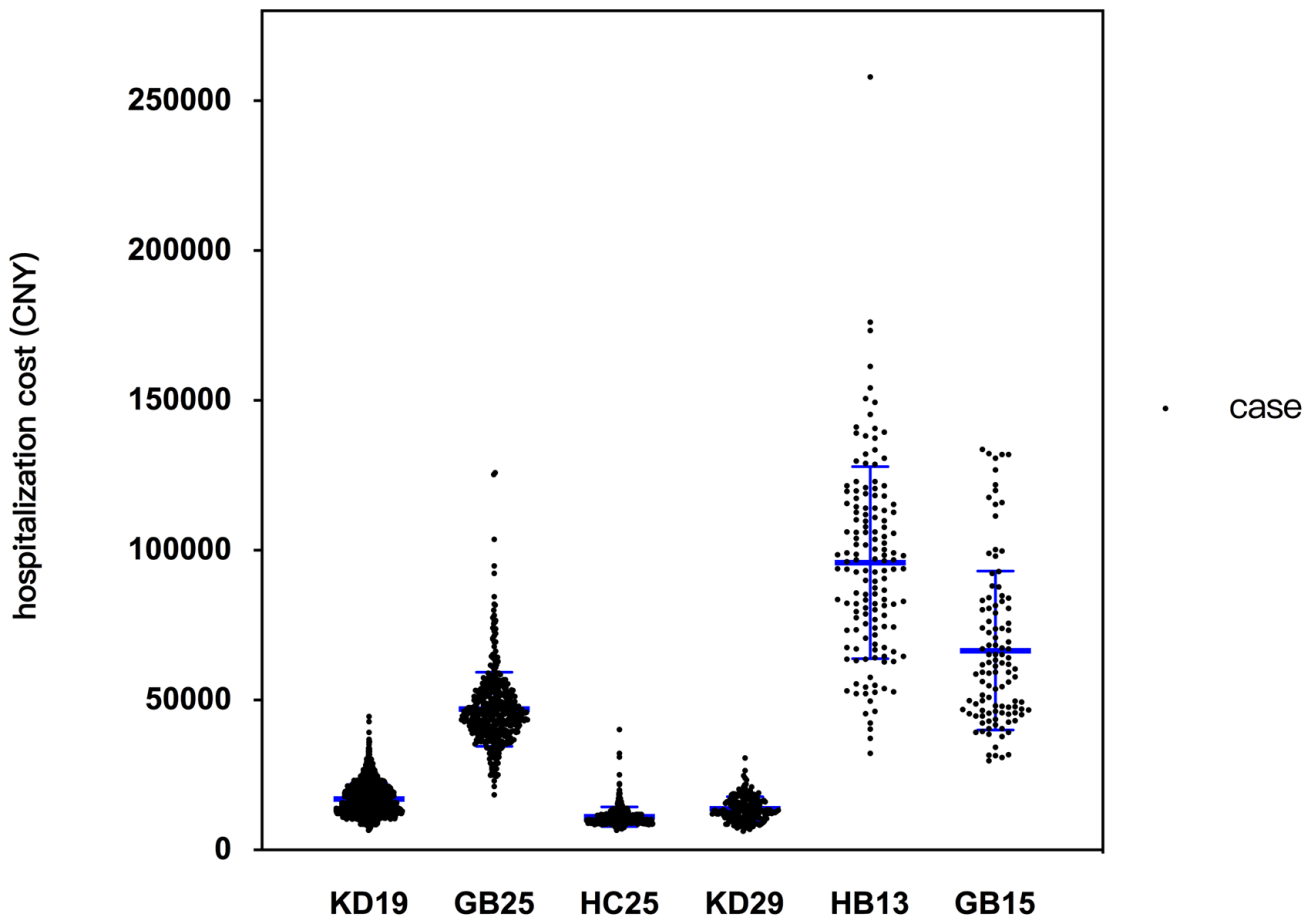
Scatter plot illustrating cost distribution of studied DRG groups. The name of DRG groups: KD19, thyroid operation; GB25, major operation of intestine and colorectum, without complications or comorbidities; HC25, cholecystectomy, without complications or comorbidities; KD29, operation of parathyroid and thyroglossal duct; HB13, major operation of pancreas and liver, with general complications or comorbidities; GB15, major operation of esophagus and stomach, without complications or comorbidities.

After preprocessing and discarding the wrong data, the final dataset included 4,387 cases, fell into 124 DRG groups. The average RW was 1.73 and the average hospitalization cost was 25381.93 CNY. Detailed cost information of DRG groups with more than 100 cases is presented in Table 1, and a scatter plot illustrating cost distribution is shown in Figure 2. The CVs of HB13 and GB15 are higher than those of other groups. HB13 was selected for further analysis of factors influencing hospitalization cost. GB15 was not selected due to its relatively limited number of cases, which makes it difficult to perform a robust statistical analysis on the entire dataset or its subgroups.

**Table 1:** Cost information of DRG groups with more than 100 cases in department of general surgery in author’s institution.

DRG group	Number of cases	RW	Payment standard	Average cost	Min. cost	Max. cost	SD	CV
KD19	1886	1.09	22,266	17095.81	6559.20	44502.00	5215.06	0.30
GB25	440	4.26	87,110	46981.75	18365.47	125867.90	12375.01	0.26
HC25	357	0.84	17,092	11112.68	6545.36	40212.35	3275.84	0.29
KD29	185	1.02	20,919	13729.74	6328.94	30772.07	4129.09	0.30
HB13	142	4.73	96,648	95900.02	32256.91	258037.80	38002.52	0.40
GB15	109	5.47	111,639	66530.96	29827.59	133703.40	26089.97	0.39

The name of DRG groups: KD19, thyroid operation; GB25, major operation of intestine and colorectum, without complications or comorbidities; HC25, cholecystectomy, without complications or comorbidities; KD29, operation of parathyroid and thyroglossal duct; HB13, major operation of pancreas and liver, with general complications or comorbidities; GB15, major operation of esophagus and stomach, without complications or comorbidities. All costs are presented in Chinese Yuan (CNY).

Group HB13 included a total of 142 cases. Patient demographics and clinical characteristics, including gender, age, nature and location of the tumor, surgical approach, etc. are detailed in Table 2. For malignant and borderline tumors located in the head or uncinate process of the pancreas, pancreaticoduodenectomy (Whipple procedure) is commonly performed, while tumors in the body or tail of the pancreas are subjected to distal pancreatectomy. Surgical approach for tumors in the pancreatic neck is determined based on intraoperative findings. For benign tumors, local excision may be performed if the tumor volume is small and the main pancreatic duct is not involved. Depending on patient preference, surgeon experience and habits, either laparoscopic or robotic-assisted surgery may be employed. In cases where preoperative assessment indicates a large tumor involving surrounding vital organs or large vessels, or intraoperative anomalies, such as adhesions or bleeding that impede laparoscopic maneuvers, open surgery is pursued.

**Table 2:** Demographic and clinical characteristics of HB13 cases.

		Number of cases	Proportion (%)
Gender	Male	67	47.2
	Female	75	52.8
Age	<45	37	26.0
	45–59	33	23.2
	60–75	65	45.8
	>75	7	4.9
	Median: 60; Mean: 58.3; SD: 15.0		
Location of tumor	Head	37	26.0
	Neck	13	9.2
	Tail/body	67	47.2
	Ampulla	25	17.6
Nature of tumor	Benign	27	19.1
	Malignant	108	76.0
	Borderline	7	4.9
Surgical approach	Laparoscopic	66	46.4
	Robotic	62	43.7
	Open	14	9.9
Procedure	Pancreaticoduodenectomy	60	42.3
	Distal pancreatectomy	72	50.7
	Local excision and others	10	7

**Figure 3:**
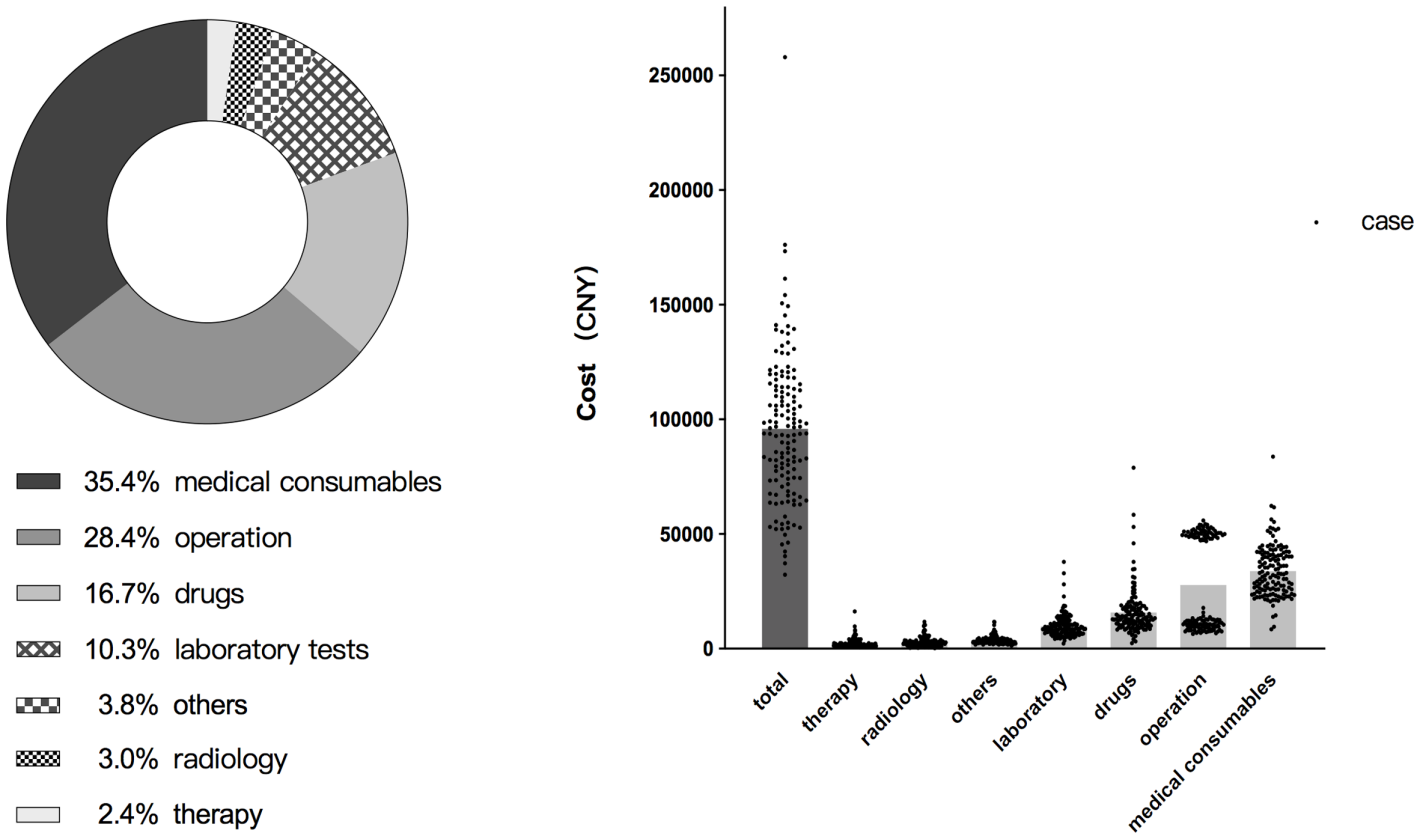
Proportion and cost distribution for each service category of HB13 patients.

The total cost of hospitalization can be divided into seven service categories (from highest proportion to lowest): medical consumables, operation, drugs, laboratory tests, others, radiology, and therapy. Details of the cost for each category are shown in Table 3 and Figure 3.

**Table 3:** The cost information for each service category of HB13 patients.

	Average	Min.	Max.	SD
Total cost	95,900	32,257	258,038	38,002
Therapy	2,259	775	16,196	1951
Radiology	2,891	294	11,676	2094
Others	3,656	1,397	11,791	1726
Laboratory	9,849	2,226	37,913	5,066
Drugs	16,037	2,389	78,994	10,147
Operation	27,241	6,476	55,900	19,824
Consumables	33,984	8,412	83,694	11,013

### 3.2 Semi-structured interview results

**Figure 4:**
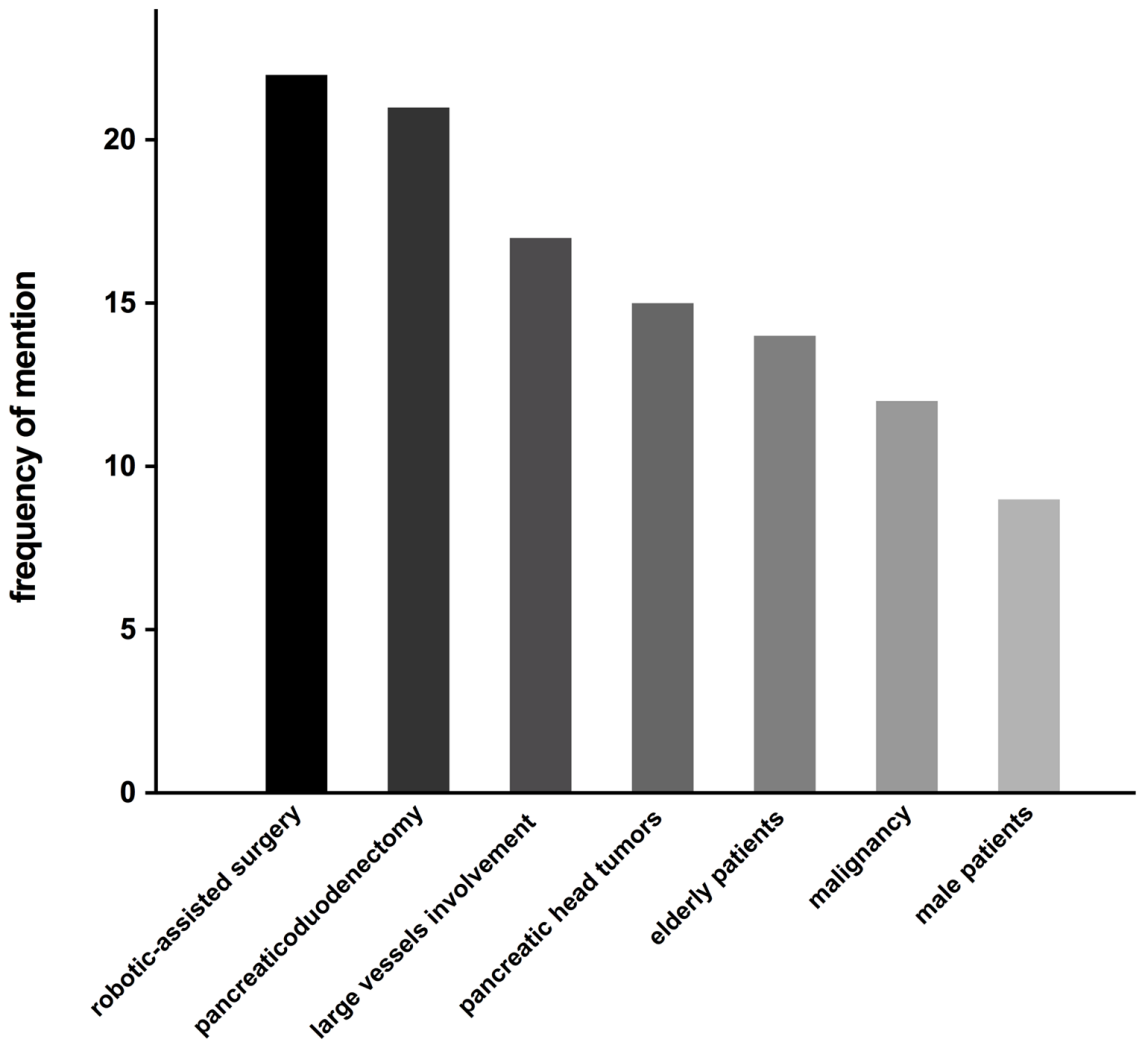
The frequency of factors mentioned as potential contributors to increased hospitalization costs.

The semi-structured interview, designed in attempt to identify potential clinical factors influencing the hospitalization cost of pancreatic surgery patients, was administered to 22 pancreatic surgeons in author’s department. 17 respondents stated that the current grouping scheme for pancreatic surgery patients fails to meet the needs of clinical practice, while 20 respondents considered the current payment standard for pancreatic surgery patients to be below the actual cost. The clinical factors most frequently mentioned during the interviews as potential contributors to increased hospitalization costs were: robotic-assisted surgery (22/22), pancreaticoduodenectomy (21/22), involvement of large vessels (17/22), pancreatic head tumors (15/22), older adult patients (14/22), malignancy (12/22), and male patients (9/22), listed in descending order (Figure 4).

### 3.3 Multiple linear regression analyses of the cost-influencing factors

Based on the semi-structured interview results, seven clinical factors were examined via multiple linear regression to analyze their actual impact on total cost of HB13 patients, including age, gender, tumor location, tumor nature, pancreaticoduodenectomy or not, robotic-assisted surgery or not, and large vessel resection (portal vein, SMV or SMA) or not. The latter 6 variables are categorical, while age was continuous. The results showed that pancreaticoduodenectomy and robotic-assisted surgery significantly increase the total cost, with both *p*-values being less than 0.0001 (Table 4). Collinearity has been excluded from the regression results, with an R-squared value of 0.59 for the regression model. The residuals approximately follow a normal distribution as confirmed by a histogram test.

**Table 4:** Multilinear regression analysis of total hospitalization cost.

		B	|t|	*p*-value	VIF
Intercept		51,182	3.661	0.0004	-
Age		102.0	0.6731	0.5021	1.338
Gender^1^	Female	−3,030	0.7513	0.4538	1.120
Tumor location^2^	Ampulla	−1892	0.3082	0.7584	1.511
	Body/tail	7,771	0.7900	0.4310	6.073
	Neck	9,324	0.9978	0.3202	2.007
Tumor nature^3^	Malignant	2,532	0.4824	0.6303	1.387
	Border line	−1,187	0.1192	0.9053	1.285
Pancreaticoduodenectomy^4^		37,487	4.284	<0.0001	4.165
Robotic-assisted surgery^5^		41,873	10.001	<0.0001	1.192
Large vessel resection^6^		6,075	0.7059	0.4815	1.088

1 Reference level: male.

2 Reference level: head.

3 Reference level: benign.

4 Reference level: non-pancreaticoduodenectomy.

5 Reference level: non-robotic surgery.

6 Reference level: no large vessel resection.

**Figure 5:**
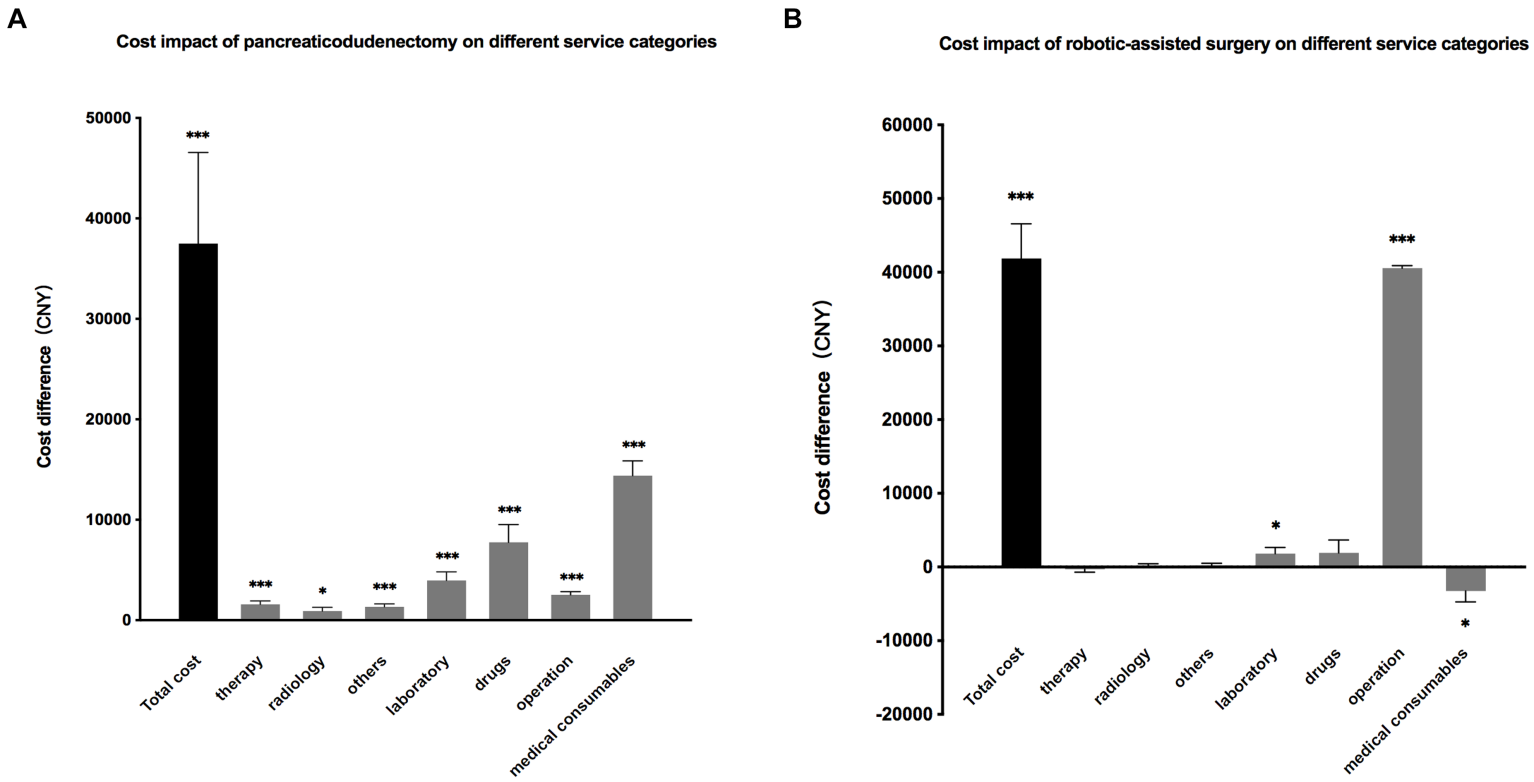
(**A**). The impact of pancreaticoduodenectomy on total hospitalization cost and the costs of seven service categories. The results are presented as the difference in costs compared to patients who did not undergo pancreaticoduodenectomy. (**B**). The impact of robotic-assisted surgery on total hospitalization costs and the costs of seven service categories. The results are presented as the difference in costs compared to patients who did not undergo robotic-assisted surgery.

Another multilinear regression was conducted to further analyze the impact of pancreaticoduodenectomy and robotic-assisted surgery on the cost of seven service categories. The results showed that patients undergoing pancreaticoduodenectomy had a significantly increased cost across all categories, with an average increase of 14,396 CNY for medical consumables, 7,764 CNY for drugs, and 3,960 CNY for laboratory tests. Patients who underwent robotic-assisted surgery had a significant increase in operation fees (40,571 CNY) and laboratory tests (1807 CNY), while experiencing a decrease of 3,267 CNY for medical consumables (Figure 5).

### 3.4 Proposal of subdividing group HB13 and its statistical validation

**Figure 6:**
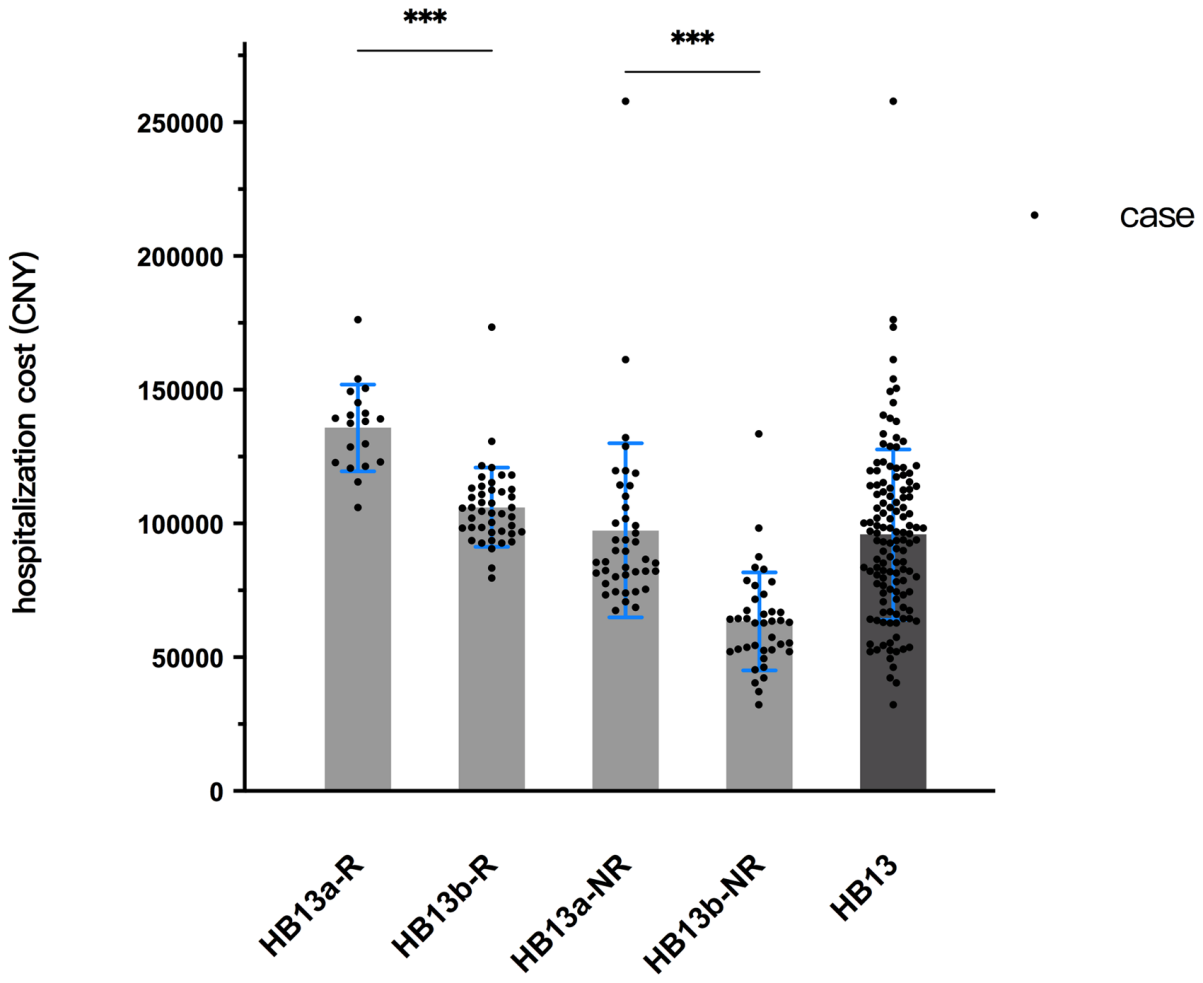
Cost distribution of subgroups of HB13.

Based on the above analysis of influencing factors, all patients who underwent pancreaticoduodenectomy were picked out separately, a t-test was employed to examine the difference in hospitalization cost between these patients and the remain of HB13. To eliminate interference from another influencing factor, robotic-assisted surgery, the original HB13 group was divided into the following subgroups: robotic surgery with pancreaticoduodenectomy (HB13a-R), robotic surgery without pancreaticoduodenectomy (HB13b-R); non-robotic surgery with pancreaticoduodenectomy (HB13a-NR), and non-robotic surgery without pancreaticoduodenectomy (HB13b-NR). The results showed that regardless of robotic surgery, patients undergoing pancreaticoduodenectomy had significantly higher hospitalization cost than those without the procedure. The CV of all 4 subgroups was lower than that of the original HB13 group. The average cost of HB13b-NR was below the current payment standard for HB13, while the average cost of the other three subgroups exceeded the current standard (Table 5; Figure 6).

**Table 5:** Cost information and *t*-test result of subgroups of HB13.

Subgroup	Number of cases	Payment standard	Average cost	Min. cost	Max. cost	SD	CV	*t*	*p*-value
HB13a-R	19	-	135,811	106,037	176,220	16,149	0.12	7.048	<0.001
HB13b-R	43	-	106,142	79,621	173,444	14,893	0.14		
HB13a-NR	41	-	97,898	67,553	258,038	30,229	0.31	5.713	<0.001
HB13b-NR	39	-	63,485	32,257	133,541	18,310	0.29		
HB13	142	96,648	95,900	32,257	258,038	38,002	0.40	-	-

## 4 Discussion

Based on experiences from Europe and the U.S, although the specific details such as grouping schemes, payment standard, and grouping evaluation criteria vary from country to country and are continuously subject to dynamic adjustments, there is one feature they share in common: the total number of DRG groups tends to increase over time (6). Having a greater number of groups reflects a more precise and refined DRG grouping scheme, which helps better differentiate patients with varying medical resource consumption, although they may share similar clinical characteristics. This leads to a higher intra-group cost homogeneity, and a more accurate payment standard for each disease. It significantly reduces the waste of health funds and medical resources caused by high intra-group heterogeneity, as well as adverse consequences such as cutting services, upcoding, and cherry-picking (5, 6, 8). To split an existing DRG group for better grouping efficacy, two conditions should be met to make it justifiable: ① high cost heterogeneity within the group; ② existence of clinical characteristics, identifiable and measurable, that explain this heterogeneity. In CHS-DRG system, the current criteria for evaluating the grouping efficacy is CV < 1. If CV of a certain group is less than 1, no further subdivision is required. However, considering the definition of CV (SD/mean), groups with high average costs may still exhibit significant cost variability, even if they meet the CV criteria, especially when compared to groups with similar CVs but lower average costs. Taking HB13 group as an example, Table 1 shows that SD of cost is 38,002 CNY, nearly twice the payment standard of the KD19 group (22,266 CNY). It can also be observed in Figure 2 that the cost distribution of HB13 is more dispersed than the preceding groups. Therefore, it is reasonable to consider that HB13 manifests certain degree of heterogeneity, and to investigate the possibility of splitting based on clinical characteristics.

Unlike FFS and DIP payment, the DRG payment system incorporates both clinical characteristics and resource consumption (3, 10). The latter is derived from statistical analyses of historical data on cost, while the former relies on clinical experiences and expert consultation (5, 10). According to the results of semi-structured interviews conducted with pancreatic surgeons in author’s institution, seven clinical factors were selected for multilinear regression analysis to elucidate their impact on hospitalization cost. The result gives two independent influencing factors: pancreaticoduodenectomy and robotic-assisted surgery. While keeping other factors unchanged, patients undergoing robotic-assisted surgery have an average increase of 41,873 CNY in total cost, which is almost entirely derived from operation fee. As a novel technology that has been widely utilized in operations on the pancreas, gastrointestinal tract, liver and other abdominal organs, robotic-assisted surgery is well acclaimed for its advantages such as lower blood loss, lower rate of conversion to laparotomy, more lymph nodes harvested, fewer postoperative complications and reduced use of medical consumables (11–14). But its utilization, as well as that of other new drugs and therapies, which are always costly, is not favored under DRG payment where hospitals are reimbursed a fixed amount based on diagnosis and procedure, and are reluctant to be exposed to financial risk. Most Western countries with DRG payment system have developed mechanisms, such as separate payments, supplementary payments, special funding, RW or grouping adjustments, to account for technological innovations and to ensure that patient access to quality-enhancing, albeit cost-increasing, technologies is not compromised (6, 15). Since the cost increase associated with robotic surgery primarily stems from the operation fee, establishing a supplementary payment mechanism above the standard payment is recommended to mitigate the hospital’s financial risk and promote its clinical use.

Pancreaticodudenectomy is the standard procedure for malignancies in pancreas head, Vater’s ampulla, and benign lesions in these locations with main pancreatic duct involvement. While keeping other factors unchanged, patients undergoing pancreaticoduodenectomy have an average increase of 37,487 CNY in total cost, derived from all seven categories of hospital services, which is exactly in line with clinical experience. As compared to local resection, enucleation and distal pancreatectomy, pancreaticodudenectomy is the most complex pancreatic procedure with larger extent of tissue excision and multiple anastomoses of the digestive tract, which consumes great amount of high-value consumables such as absorbable suture, anastomosis stapler and endoclip, leading to significant cost increase in medical consumables. The procedure is characterized by technical difficulty and prolonged operation time, resulting in higher operation fees. Hence patients undergoing this procedure are at a higher risk of complications such as bleeding, pancreatic fistula, bile leak, and intra-abdominal infection, which contribute to extended length of stay, increased cost in drugs, laboratory tests, radiology and therapies, and a further increase in medical consumables and operation fees if a second surgery is performed to address these complications. In this context, given the discrepancies in total cost and costs for different service categories, patients undergoing pancreaticoduodenectomy and those undergoing other pancreatic procedures constitute two distinct groups, each with different clinical characteristics and resource consumptions, respectively. The point is further verified by t-test between these two groups, after eliminating the influence of robotic-assisted surgery. In this light, a new DRG group exclusively for patients undergoing pancreaticoduodenectomy should be established from the original HB13, with a higher payment standard set in accordance with the actual cost level of these patients. In this manner, we could achieve better intra-group homogeneity, improve the allocation and economization of health insurance funds, and reduce the risk of cherry-picking and compromising quality that exist under the current grouping and payment scheme, which are detrimental to patients requiring pancreaticoduodenectomy.

There are some limitations to this study. Given that DRG payment has been implemented in Beijing for just over 2 years, there is a relatively small number of cases in the HB13 group. Therefore, increasing the number of cases is necessary to further validate the reliability of the results. Moreover, the HB13 group is defined as “major operations of pancreas and liver, with general complications or comorbidities.” Due to the clinical scope of the authors’ department, liver surgery cases were not included in this study. Nonetheless, we believe this is the first statistical evaluation of the efficacy of DRG grouping for pancreatic surgery cases from a clinical perspective. To our knowledge, there are no existing studies of a similar nature that focus on pancreatic surgery or other general surgery cases. Some published articles have shown that certain procedures or techniques, such as pancreaticoduodenectomy or the robotic-assisted surgery, impact hospitalization costs, which aligns with some of our findings (14, 16, 17). But none of these studies have any implication for the DRG system evaluation or payment policy. Therefore, this study is expected to provide valuable insights for policymakers responsible for the payment system and may offer methodological guidance for exploring the possibility of adjusting current DRG groups that fail to meet clinical demands.

## 5 Conclusion

The HB13 group exhibits a relatively higher CV of hospitalization cost, which indicates a lower intra-group homogeneity. The robotic-assisted surgery and pancreaticoduodenectomy are two independent factors that significantly impact total cost. Patients undergoing robotic surgery experience an average increase of 41,873 CNY in total costs, primarily derived from operation fees. Therefore, a supplementary payment set on top of the current payment standard of HB13 should be introduced to guarantee patients access to this advanced technology. Patients undergoing pancreaticoduodenectomy experience an average increase of 37,487 CNY in total cost, with significant increases across all service categories. Therefore, a new DRG group with a higher payment standard should be established specifically for these patients. It is recommended to establish a tiered CV value based on the mean costs of different disease groups for grouping efficacy evaluation: the higher the mean cost, the lower the CV value. This approach enhances intra-group homogeneity and promotes better utilization and allocation of health insurance funds while avoiding unintended negative outcomes, such as service cuts and cherry-picking. A well-functioning DRG system should excel at creating more groups to achieve higher intra-group homogeneity, which, however, may increase the complexity of database maintenance and management. Therefore, a careful balance must be maintained between achieving cost homogeneity and keeping a manageable number of groups for archiving, comparison, and payment purposes.

## Data Availability

The original contributions presented in the study are included in the article/supplementary material, further inquiries can be directed to the first author.
